# STEFF: Spatio-temporal EfficientNet for dynamic texture classification in outdoor scenes

**DOI:** 10.1016/j.heliyon.2024.e25360

**Published:** 2024-02-05

**Authors:** Kaoutar Mouhcine, Nabila Zrira, Issam Elafi, Ibtissam Benmiloud, Haris Ahmad Khan

**Affiliations:** a MECAtronique Team, CPS2E Laboratory, National Superior School of Mines Rabat, 10080, Morocco; b ADOS Team, LISTD Laboratory, National Superior School of Mines Rabat, 10080, Morocco; c Laboratory of Conception and Systems (Electronics, Signals, and Informatics), Faculty of Science, Mohammed V University, Rabat, 10102, Morocco; d Agricultural Biosystems Engineering Group, Wageningen University & Research, Wageningen, the Netherlands; e Data Science, Crop Protection Development, Syngenta, the Netherlands

**Keywords:** STEFF, Dynamic texture, Outdoor scene classification, Deep learning, CNN, EfficientNet, Spatio-temporal features

## Abstract

In recent years, dynamic texture classification has become an important task for computer vision. This is a challenging task due to the unknown spatial and temporal nature of dynamic texture. To overcome this challenge, we investigate the potential of deep learning approaches and propose a novel spatio-temporal approach (STEFF) for dynamic texture classification that combines the representation power of motion and appearance using the difference and average operators between video sequences. In this work, we extract deep texture features from outdoor scenes and integrate both spatial and temporal features into a pre-trained Convolutional Neural Network model, namely EfficientNet, with a fine-tuning and regularization process. The robustness of the proposed approach is reflected in the promising result when comparing our method to the proposed architectures and other existing models. The experimental results on three datasets demonstrate the effectiveness and efficiency of the proposed approach. The accuracy percentages are 95.95%, 94.09%, and 98.01% on the outdoor scenes of Yupenn, DynTex++, and Yupenn++ datasets, respectively.

## 1 Introduction

Dynamic Texture (DT) has played an important role in texture analysis and attracted extensive attention in computer vision over the past few years. DT describes the spatial repetition and temporal variation that characterize video sequences, such as the motion and appearance of waves, sky clouds, windmill farms, etc. Doretto et al. [1] defined the DT by extending the principle of static textures in images from the spatial domain to the spatio-temporal domain, and they affirmed that “Dynamic textures are sequences of images of moving scenes that exhibit certain stationarity properties in time; these include sea-waves, smoke, foliage, whirlwinds, etc.” This significant progress has been made in a variety of domains, including video segmentation [2], Human Machine Interaction (HMI), video retrieval [3], or automatic facial expression recognition [4].

Temporal texture recognition contributes to many applications in real-world, including security, medical image analysis, surveillance, fire detection [5], hyper-spectral image classification [6], remote sensing, scene classification [7], automatic micro-expression apex spotting [8], [9], face spoofing detection [10], [11], [12], [13], [14], face recognition [15], face presentation attack detection [16], moving object identification [17], facial expression detection [18], 3D mask attack detection [19], and so on.

The problem and the challenge in dynamic texture classification is to correctly identify and categorize video or image sequences that display temporal fluctuations, and complex motion patterns. These sequences are characterized by their spatial and temporal nature. Traditional techniques, which largely concentrate on static images, might not be suitable for processing dynamic textures and could have limitations in capturing complex spatial and temporal characteristics. Consequently, the issue can be summed up as follows: How can we create reliable and effective methods that can successfully extract and evaluate the spatio-temporal features of dynamic textures to enable accurate classification and recognition?

In general, solving the dynamic texture classification problem is essential for maximizing the utility of dynamic texture data, enhancing real-world applications, learning about temporal phenomena, understanding the behaviors present in the data, increasing automation and efficiency, creating novel approaches, models, and algorithms that advance these domains and developing the knowledge in computer vision and related fields. It is thus necessary to overcome this critical problem. Our proposed approach combines the power of motion and appearance by utilizing the difference and average operators applied to video sequences, and then it incorporates deep learning techniques by extracting deep texture features from outdoor scenes. These features are integrated into a pre-trained Convolutional Neural Network (CNN) model called EfficientNet making it a suitable choice for dynamic texture classification. The model is fine-tuned, regularized, and evaluated to emphasize the effectiveness and novelty of the proposed method.

Zao et al. [20] explained that the major difficulties facing DT recognition are centered on the combination of spatial and temporal indicators and the extraction of defined features that are insensitive to illumination changes, robust against image transformation like rotation, and resistant to noise. Moreover, the multi-resolution analysis and the computation simplicity are considered also two challenging issues in DT recognition, when it is about searching for high efficiency.

Feature extraction is the most critical step in scene classification [21], [22]. This is mainly due to the typical combinations of motion and appearance features that contain global and local information [23]. Thus, the performance of the classifiers can be improved by having features that are specified. There are handcrafted features that provide global information, while deep learning features often extract local information [24]. The handcrafted approaches have been implemented for decades and were found to be effective tools when combined with machine learning classifiers. Indeed, these methods have been used to provide features that estimate the quality; some of them are based on structural information, while others are focused on statistical characteristics. In addition, the handcrafted features take into consideration the intensity values, the topological structure, and texture information that are often extracted during the pre-processing image step [25]. They are usually divided into appearance-, geometric-, and sequence-based features, including the Local Binary Pattern (LBP) [20], Gabor filter bank, Scale Invariant Feature Transform (SIFT) [26], SIFT-LBP [27], Wavelet Transform (WT), Grey Level Co-occurrence Matrix (GLCM), the spatial feature-based methods GIST, Histogram of Oriented Gradients (HOG) [28], Haar-like features [29] and Gabor-wavelet features [30], Gabor Texture [31], and pixel density [32].

Deep Learning (DL)-based methods have progressed rapidly in recent years. These approaches represent the deep neural network and other techniques using the powerful concept of transfer learning from pre-trained models with the fine-tuning and regularization process. They are inspired by the human brain, considering multiple signals as input, combining them linearly, and then treating them by a non-linear operation to generate an output signal. This basic process is followed by a layered architecture learned from the training phase with a sufficiently large knowledge database to make an intelligent prediction. DL algorithms showed exciting solutions and growing performance in real-world applications, such as medical imaging analysis, object detection, face identification, speech recognition, and Natural Language Processing (NLP), etc. Indeed, CNN is extremely able to extract effective and useful features, able to classify a large-scale image [33], and capture the complex non-linear interactions among them [34]. There exist several architectures such as Restricted Boltzmann Machines (RBM), Recursive Neural Networks (RNNs) [35], Long Short Term Memory (LSTM), and CNN-based methods [36], including AlexNet [37], googleNet [38], ResNet [39], DenseNet [40], EfficientNet [41], Inception V3 [42], VGGNet [43], and SqueezeNet [44].

Therefore, Chetouani et al. [24] affirmed that combining DL and handcrafted features showed promising results in performance, compared to existing methods that used only one kind of solution for the extraction step. This improvement is mainly due to the diversity of the information and the robustness of the features extracted. In this paper, our contribution is following up this scientific research very closely, the idea consists of developing a new architecture based on handcrafted and deep learning features, employing the spatio-temporal concept to extract robust, global, and local features, generating the best classification rate with optimal benefits.

The remainder of this paper is represented as follows. Section 2 represents the literature review, discussing the local spatial and spatio-temporal approaches for dynamic texture classification. Section 3 introduces the proposed STEFF approach and describes the steps involved in our novel methodology; data pre-processing, transfer learning, fine-tuning, and regularization of data are also covered in Section 3. Section 4 represents the model tuning parameters, hardware implementation, and performance evaluation metrics of our proposed architecture. Section 5 shows the experiment-based evaluation of the proposed approach on outdoor scene datasets: Yupenn, Dyntex++, and Yupenn++. Results and discussion are also presented in Section 5. Finally, conclusions and perspectives are provided in Section 6.

## 2 Literature review

During the past decades, studies have developed a large number of dynamic texture classification methods using the representational power of deep learning [45]. Among them, we find local spatial-based approaches that focus on features extracted by local descriptors like the Local Binary Pattern (LBP) extension, shedding light on appearance information. Along with this solution, there are spatio-temporal approaches that work with the concept of time by analyzing the movements and gestures that emerge from a given scene.

### 2.1 Local spatial-based approaches

Yang et al. [46] addressed the problem of dynamic texture classification by gathering the spatial and temporal texture features via an ensemble SVM architecture. The dynamic texture video is transformed into static textures to benefit from the spatial texture features of a single frame, and several frames of the DT video are randomly selected in the time augmentation process. The naïve Linear Dynamic System (LDS) model is used to extract dynamics from DTs in the temporal domain. Nguyen et al. [47] invented an efficient DT representation called Difference of Derivative Gaussians (DoDG), which is a filtering kernel resistant to noise for DT. They constructed discriminative DoDG-based descriptors in small dimensions using one of the LBP variations to extract local features, which is particularly useful for mobile. Nguyen et al. [48] proposed the Hierarchical Local Pattern (HILOP) by focusing on the connections between a pair of regional hierarchies instead of those between a center pixel and its local neighbors, as done in LBP versions. Furthermore, it is possible to add more potent discriminative information by integrating the hierarchical features acquired in multi-supporting hierarchies. Yeo et al. [49] presented a scene classification algorithm based on semantically segmented objects, which allows the detection of objects in the sequence. They created a weight matrix with bias values to determine a scene class statistically. Then, they classified the image by using the constructed weighting matrix. Wu et al. [50] presented forest fire recognition based on feature extraction from multi-view sequences. Specifically, a Graph Neural Network (GNN) model based on the feature similarity of multi-view images was proposed. Thereafter, a dynamic feature extraction method was designed using fire area segmentation to extract the key features from images. Nguyen et al. [51] proposed the Momental Directional Patterns (MDP) for dynamic texture classification. They considered the approaches based on local features and the filtering process, using global features extracted from max-pooling videos to form an effective and discriminative descriptor. Previtali et al. [52] applied Dynamic Mode Decomposition (DMD) and Dynamic Mode Decomposition with Control (DMDC) for dynamic texture identification. Moreover, they compared the results to those of classical approaches from mathematical and computational points of view. Ma et al. [7] invented the SceneNet approach, which uses multi-objective neural evolution architecture search to classify remote sensing scenes in deep learning networks, which is an optimization method. An evolutionary algorithm is used to code and search a network architecture, which can create a more dynamic hierarchical extraction of the remote sensing image scene features. Giveki et al. [27] presented a new method for scene classification using a feature integration method using Scale Invariant Feature Transform (SIFT) and LBP. Also, they presented a new framework for training a radial basis function neural network, combining the optimum steepest descent method with a Particle Swarm Optimization (PSO) based artificial neural network classifier. Sinha et al. [53] suggested a new color GPHOG descriptor to enhance the Pyramid of Histograms of Oriented Gradients (PHOG) descriptor for object and scene image classification, which encodes information from color, shape, spatial, and local aspects of an image.

### 2.2 Spatio-temporal-based approaches

There exist other different types to solve this kind of problem, and a large portion will go to the dynamic sector. Spatio-temporal-based approaches are constructed to describe the motion and spatial architecture of each local neighborhood of textures.

Chaos+GIST is a spatial and temporal feature-based approach proposed by Shroff et al. [54]. They used the theory of chaotic systems to capture dynamics. The Histograms of Optical Flow (HOF) combined with GIST represented a spatio-temporal method, which is applied for DTs classification [55], [54], [56]. Zhao et al. [57] introduced the Volume Local Binary Pattern (VLBP) method to extend the Local Binary Pattern descriptor to the spatio-temporal domain. The size of VLBP texture features increases rapidly with the number of neighboring pixels, despite their robustness and insensitivity to rotation and monotonic gray-scale changes. Due to this exponential growth, applying VLBP to a large framework becomes computationally intensive. For this reason, Zhao et al. [20] proposed the Local Binary Pattern in Three Orthogonal Planes (LBP-TOP), which reduces the dimension of the VLBP feature descriptor. In [58], a 2-D Histogram Fourier LBP-TOP (2DHFLBP-TOP) was proposed which effectively worked with rotation variations of DTs, and was robust with respect to changes in viewpoint. Nanni et al. [59] proposed Local Ternary Patterns from Three Orthogonal Planes (LTP-TOP), where they combined the idea of LBP-TOP with Local Ternary Patterns (LTP). Ribas et al. [35] presented a learning graph representation with a randomized neural network. Additionally, they used a developed representation from the RNN model, applying one-parameter directed spatio-temporal graph modeling (i.e., radius) to describe motion and the appearance of the DT. Ali et al. [60] proposed a Maximum Posteriori Approximation (MPA) of Hidden Markov Models (HMM) for proportional sequential data modeling with simultaneous feature selection. The approach achieved an accuracy of 93.33%. Yang et al. [61] proposed a spatio-temporal Generative Adversarial Network (GAN) based on the DT synthesis method for surveillance video coding. They employed the 3D convolutional layer and a spatio-temporal discriminator to explore spatial and temporal information. Yao et al. [62] proposed a remote photoplethysmography (rPPG) based spoofing detection approach for face mask attacks using the EfficientNet model on a weighted spatial-temporal map. They solved the distorted signal problem generated by background noise or object motion. In this way, various regions of interest covering the entire face and containing rich rPPG signals are enhanced, forming a weighted spatial-temporal map. Bonomi et al. [10] developed the Local Derivative Patterns on Three Orthogonal Planes (LDP-TOP), which contributes to the analysis of spatio-temporal texture dynamics of the video signal to distinguish between real and fake sequences. Esmaeili et al. [9] proposed LBP from Six Intersection Planes (LBP-SIP) for automatic micro-expression apex frame spotting. This method extracted LBP code from six intersection planes and then combined them. Moreover, it could be applied in situations where the detection of small variations is required. Zheng et al. [63] proposed Dynamic Texture Fusion (DTexFusion) using a consumer RGBD sensor, which is resistant to noise, distortions, and mistakes in the predicted object motion due to color and depth input issues. Chen et al. [64] presented extracted dynamic textures and changes in facial configuration for video-based facial emotion detection with a new feature descriptor termed the Histogram of Oriented Gradients from Three Orthogonal Planes (HOG-TOP) and a new geometric feature descriptor. Nguyen et al. [65] proposed an efficient model for DT description using Gaussian-based filtering to extract blurred-invariant features from a DT scene and create a Local Rubik-based Pattern (LRP) operator to capture appearance and motion characteristics. Finally, they provided a thresholding/encoding technique to extract detailed spatio-temporal relationships from a Rubik's cube to construct a robust descriptor resisting environmental changes. Torabian et al. [5] developed a fire detection algorithm based on motion analysis using spatio-temporal features as correlation coefficient and applying kernel Principal Component Analysis (PCA) technique with fractal analysis. Zhou et al. [12] proposed an effective face anti-spoofing method based on dynamic color texture analysis, using the Local Directional Number pattern (LDN) with a derivative-Gaussian mask to record finely detailed appearance information while resisting noise and illumination changes. Therefore, to capture motion information, the LDN was extended to a spatial-temporal variant named the Local Directional Number pattern from Three Orthogonal Planes (LDN-TOP). Xu et al. [66] proposed a deep neural network architecture combining LSTM units with CNN for attack detection. Huang et al. [67] proposed Long Short Term Features (LSTF), where the Short-Term Deep Features (STDF) are combined with Long-Term Frequency Features (LTFF) that were extracted using the autoregressive moving average model. Zhao et al. [68] proposed a dynamic texture classification approach, using unsupervised 3D filter learning and local binary encoding. Therefore, they extracted local binary features from the spatio-temporal domain with 3D filters. Luo et al. [69] proposed a dynamic texture feature named LBP on the TOP and GLCM Histograms (LTGH) for working condition recognition in the froth flotation, which applied the LBP and Gray-Level Co-occurrence Matrix (GLCM) histograms on the TOP. Rivera et al. [70] introduced a dynamic-micro-texture descriptor, i.e., a spatio-temporal Directional Number transitional Graph (DNG), which describes both the spatial structure and motion of each local neighborhood by capturing the direction of natural flow in the temporal domain. Yang et al. [46] proposed E-SVM, where they addressed the problem of dynamic texture recognition in a simple and general way to aggregate spatial and temporal texture features via an ensemble SVM scheme. Tavakolian et al. [71] designed a Deep Discriminative Model (HDDM) using Gaussian Restricted Boltzmann Machines (GRBM) to initialize parameters. The spatio-temporal variation patterns within frames are extracted and represented sparsely using the Sparse Cubic Symmetrical Pattern (SCSP). Hong et al. [72] proposed a codebook-based DT descriptor that aggregates salient features on three orthogonal planes (ASF-TOP). This process removes the feature from outlier frames that suddenly or rarely appear in a particular context, thus enhancing the emphasis of the salient features. Ren et al. [73] proposed the PCA-cLBP/PI-LBP/PD-LBP, using the Principal Histogram Analysis on the covariance matrix of the LBP histograms (PHA-LBP) to remove the unreliable information. The process can be derived in a patch-independent manner or a patch-dependent manner, PI-LBP, and PD-LBP. Theriault et al. [74] learned motion descriptors with Slow Features Analysis (SFA) which represents the principal and more stable motion components of training videos. Feichtenhofer et al. [75] proposed Bags of Spacetime Energies (BoSE), which is built on primitive features that uniformly capture the spatial and temporal orientation structure of the imagery. They proposed also the Dynamically Pooled Complementary Features (DPCF) [76] that analyzed a dynamic scene in terms of spatial, temporal, and color characteristics. Mumtaz et al. [77] introduced the Bag of Systems Trees (BoST), which describes the motion patterns in spatio-temporal patches extracted from the video. Ahonen et al. [78] proposed the Local Phase Quantization (LPQ) based on quantizing the Fourier transform phase in local neighborhoods, and applied it on three orthogonal planes to extract the motion from dynamic appearance, constructing, in the end, the LPQ-TOP [79]. Feichtenhofer et al. [80] aggregated complementary information from separate spatial and temporal orientation measurements in spacetime pyramids via a random forest classifier (CSR). Vasudevan et al. [56] proposed SIFT+5DMFV that introduces a five-dimensional feature vector extracted from the optical flow field, employing then the Spatial Pyramid Matching (SPM) algorithm on combined SIFT [81] descriptor and motion feature descriptor to perform classification. Konda et al. [82] proposed the Synchrony Autoencoder (SAE) approach, showing that learning about synchrony is possible using very fast local learning rules and can be viewed as performing greedy parameter estimation in some motion energy models. Uddin et al. [83] introduced the Directional Local Ternary Pattern from Three Orthogonal Planes (DLTP-TOP), using Apache Spark to conduct distributed computing for large-scale data, and classifying with a CNN algorithm.

### 2.3 Dynamic-based approaches

Outdoor scene recognition is a supervised machine learning problem and a classification task that has various applications in different domains, such as autonomous flight drones, autonomous driving, monitoring cameras, image processing, image retrieval, etc., which indicates the importance of this task and the necessity to develop an efficient and suitable algorithm to generate robust models. The proposed methods were grouped into six categories [47], [84].

#### 2.3.1 Model-based methods

Model-based methods describe and simulate the troubled behavior of dynamic textures. Wang et al. [85] introduced a chaotic vector approach to extract chaotic features from each pixel intensity series in a video.

#### 2.3.2 Filter-based methods

Filter-based methods have been applied to texture recognition. Zhao et al. [86] had promising results in DT classification using Multiscale PCA-learned Filters from image sequences on Three Orthogonal Planes (MPCAF-TOP). Meanwhile, learned filters have been addressed to construct DT descriptors, e.g., Multiscale Binarized Statistical Image Features on Three Orthogonal Planes (MBSIF-TOP) [87]. B3DF_SMC [68] used unsupervised 3D filter learning and local binary encoding. Rivera et al. [70] introduced a dynamic micro-texture descriptor, i.e., spatio-temporal directional number transitional graph using a plane mask (DNGP). It describes both the spatial structure and motion of each local neighborhood by capturing the direction of natural flow in the temporal domain.

#### 2.3.3 Geometry-based methods

Geometry-based methods represent the appearance information of DTs, which are usually based on fractal analyses. Xu et al. [88] proposed a typical Dynamic Fractal Spectrum (DFS), Volumetric DFS (V-DFS), Multi-slice DFS (S-DFS), and its crucial extension, called Multi Fractal Spectrum (MFS) [89]. The analyses of wavelet were based on the spatial frequency of two complementary wavelet pyramids (standard multiscale and wavelet leader) and fractal patterns were addressed for DT representation. Then, they improved this problem by taking spatial information into account to construct a Wavelet-based Multi Fractal Spectrum (WMFS) for DT recognition. Fractal analysis is also employed by 3D Oriented Transform Feature (3D-OTF) [90]. Accordingly, Chen et al. [64] proposed the HOG from Three Orthogonal Planes (HOG-TOP), based on the dynamic appearance and geometric features. Quan et al. [91] proposed a method of Spatio-Temporal Lacunarity Spectrum (STLS) to encode the stationary irregularities by lacunarity-based features of local binary patterns distributions in DT, which has strong robustness to monotonic illumination changes, viewpoint changes, and classification. In the meanwhile, Baktashmotlagh et al. [92] used the subspace analysis to capture the stationary part of the video signal, to generate low dimensional feature descriptors named Kernel Stationary Subspace Analysis (KSSA), Discriminative Kernel Stationary Subspace Analysis (DKSSA), and Nonlinear Stationary Subspace Analysis (NLSSA) for temporal texture analysis. Lately, Harandi et al. [93] developed the Kernelised Grassmann Dictionary Learning (KGDL). Yuhui Quan et al. proposed the Orthogonal Tensor Dictionary Learning (OTDL) [94] which is very fast and more scalable to high-dimensional data. They also proposed the Equiangular Kernel Dictionary Learning (EKDL) [95] to exploit the nonlinear sparsity of high dimensional visual data.

#### 2.3.4 Optical-flow-based methods

Optical-flow-based methods have represented DTs based on directions and magnitudes of normal flow. Nguyen et al. [96] proposed the Features of Directional Trajectory (FDT) and their Motion Angle Patterns (FD-MAP) to exploit local features and motion information from dense trajectories, using Local Vector Patterns (LVP) in full direction on three orthogonal planes. Lately, they introduced Directional Dense Trajectory Patterns (DDTP) [97], to construct dense trajectory-based descriptors with more robustness, using spatio-temporal features of their motion points.

#### 2.3.5 Local-feature-based methods

Local-feature-based methods have described the local structure of DTs, using the simple computation of the Local Binary Pattern and its variants. [20], [98], [48], [99], [100], [86], [101], [102] Nguyen et al. [103] developed a robust approach against noise: the Completed and Statistical Adaptive Patterns on Three Orthogonal Planes (CSAP-TOP). It considers the impact of high-order filtered images and adaptive thresholding in Volume Statistical Adaptive Patterns (V-SAP), which have a small feature size compared to Completed Volume Local Binary Patterns (CVLBP) [84]. They also constructed the Momental Directional Patterns $\left(MMDP_{D\_M/C}\right)$, $\left(MEMDP_{D\_M/C}\right)$ [51]. There are different approaches for efficiently representing DTs such as local Rubik Gaussian based patterns (RUBIG) [65], to address some issues such as illumination, noise, and changes of environments, scales negatively that impact the chaotic motion of DTs. Moreover, Volumes of Blurred-Invariant Gaussians (V-BIG) [104] and Multiresolution Edge Weighted Local Structure Pattern (MEWLSP) [105] are addressed for DTs recognition. In the meantime, the gradients of Gaussian kernels were exploited in [106] for High-order 2D/3D Gaussian-gradient-based Features $\left(HoGF^{2D/3D}\right)$ descriptors, and also the Difference of Derivative Gaussians (DoDG) [47] kernel for the filtering.

#### 2.3.6 Learning-based methods

Ghanem et al. [107] proposed the DL-PEGASOS approach, which is a Maximum Margin Distance Learning (MMDL) method based on the Pegasos algorithm. Wang et al. [108] learned a high-level feature using a Deep Neural Network (DNN) for semantic visual feature learning in DTs recognition. Andrearczy et al. [109] proposed two approaches based on a CNN method applied on three orthogonal planes, called DT-CNN-AlexNet and DT-CNN-GoogleNet. Derpanis et al. [110] proposed Spatio-temporal Oriented Energy (SOE), whereas Hadji et al. [111] proposed a Spatio-temporal Oriented Energy Network (SOENet). Also, Tran et al. [112] proposed an effective approach for spatio-temporal feature learning using deep 3D Convolutional Networks (3D ConvNets) trained on a large-scale supervised video dataset (C3D).

In other work, Huang et al. [113] introduced the Attentive Temporal Pyramid Network (ATP-Net), which uses a temporal pyramid structure with an integrated attention mechanism to identify the informative aspects of frames that contain the most pertinent information to the scene. Qi et al. [114] developed Deep spatio-temporal structures, named Transferred ConvNet Features (TCoF), generated from a CNN implementation, i.e., AlexNet [115] for the frames of a video. Zheng et al. [116] proposed a Bi-heterogeneous CNN (Bi-CNN), based on deep learning, to extract both spatial and temporal features, which can facilitate tracking processes, object detection and increase the performance of visual surveillance. Gangopadhyay et al. [117] analyzed the performance of Statistical Aggregation (SA) techniques on various pre-trained CNN models by extracting CNN activation features for several frames in a video and then using an aggregation scheme to obtain a robust feature descriptor for the video. Hong et al. [118] proposed the Deep dual descriptor (D3), D3 using only static features from the keyframes $\left(D3_{S}\right)$ or dynamic features $\left(D3_{d}\right)$. There are also the Hybrid-CNN [117], the Single-Frame-CNN [119], and 3D-PyraNet-F [120].

In our research, we aim to accurately categorize videos and images with intricate motion patterns, such as changing speeds and complex movements. These dynamic textures, distinguished by their spatial and temporal characteristics, necessitate a departure from conventional techniques tailored for static images. Our central inquiry revolves around the crucial question: How can we develop reliable methods to extract and comprehend the distinctive features of dynamic textures, ensuring precise sorting and recognition? As we delve into the dynamic texture classification literature, the research confronts the challenge of precisely categorizing sequences with complex motion patterns. While existing studies predominantly concentrate on extracting efficient features from both static and dynamic textures, traditional techniques designed for static images may inadequately capture the intricate spatial and temporal characteristics inherent in dynamic textures. Our contribution lies in focusing on sorting sequences with complex motion patterns, synthesizing both movement and appearance aspects for efficient classification. This involves developing comprehensive approaches that consider the nuances of both the dynamic movement and visual features of dynamic textures. We achieve this by integrating movement and appearance through deep learning techniques, extracting detailed features from outdoor scenes. The resolution of the dynamic texture sorting challenge extends beyond theoretical implications, with tangible effects on understanding time-related events, deciphering details in data behavior, and enhancing automation and efficiency in computer vision and related domains.

## 3 Methodology

**Figure 1:**
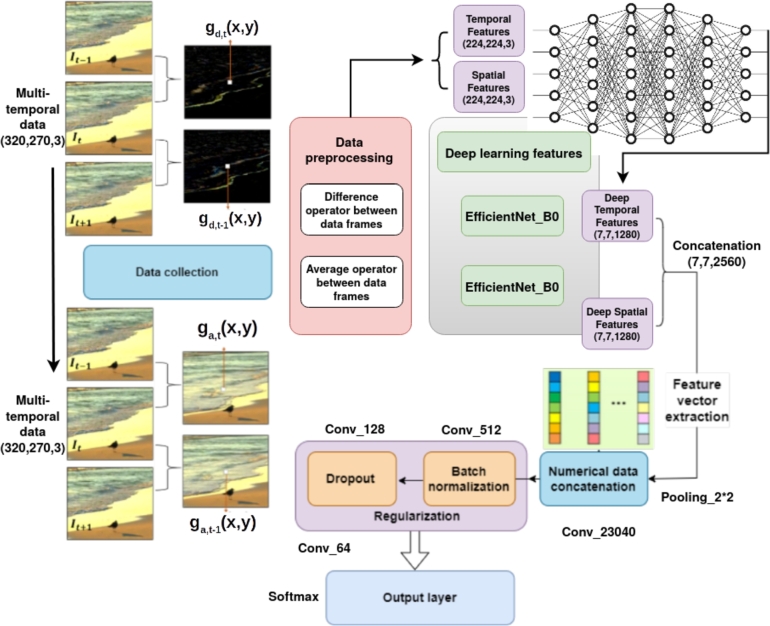
The detailed structure of the proposed STEEF approach.

In this section, we explain the different steps of our methodology, as graphically illustrated in Fig. 1. In general, the concept is based on three major phases: data pre-processing and transfer learning, with the fine-tuning process using pre-trained weights from ImageNet and network regularization.

### 3.1 Data preparation and pre-processing

Data pre-processing is the critical stage in preparing the vector data and making it suitable before it is passed to the network model. First, we have considered 3-dimensional dynamic texture videos, which are extracted and partitioned into multiple frames. We have considered 10 frames from each video. Images are in the format RGB so that deep learning models can learn useful insights from the color intensities. In this context, the data is organized and reduced by normalization in ways that make it more efficient to train the recognition model. In our proposed study, we have extracted spatio-temporal features, which are described in detail in the next section. These images are divided into training and test sets, resized to 224×224 pixels, and passed through the pre-trained CNN model to perform transfer learning-based training of this model in two parallel ways: spatial analysis and temporal analysis. Finally, the outputs are converted into numerical data, concatenated motion, and appearance vectors generated from our pre-trained CNN model. Then, they are regularized to improve the accuracy of the classification task. Fig. 1 describes the proposed methodology. Besides, we have used the DynTex++, Yupenn, and Yupenn++ datasets that are composed, respectively, of 14, 18, and 13 classes. Each dataset presents outdoor scenes like the movement of waves on the beach or blossoming branches in the wind. Some of these images are shown in Section 5.

In this study, our proposed methodology is based on motion characteristics to capture temporal features and appearance characteristics to generate spatial features. This incorporation provides different aspects of discriminative information in images and describes the structure of an image from various sides. Moreover, combining multiple features and extracting different types of patterns can generate complementary visual data for the semantic description of dynamic texture. These two processes of feature representation are described in the architecture presented in Fig. 1. Therefore, the proposed approach exploited the appearance characteristic of image data.

Thus, as we mentioned in the introduction section, there are many different techniques for extracting the distinctive features, used in texture analysis as the LBP and its variants, statistical methods such as the Gray Level Co-occurrence Matrix (GLCM) feature, or transform domain methods such as the Gabor filter descriptor. We have calculated the mean value at the level of the pixel data between the time points *t* and t+τ. Where *τ* is the time interval between two selected frames. This operation generates modified images with average properties that represent the intensity features and provide intrinsic spatial information. For this, consider an arbitrary pixel (x,y) in a monochrome image $I_{t}$ in instant *t* with a gray level of $g_{a,t}(x,y)$. Mathematically, spatial appearance is formulated in Equation (1):

(1) $$g_{a,t}(x,y)=\frac{1}{2}[g_{t}(x,y)+g_{t+\tau }(x,y)]$$

In addition to the spatial appearance, we have used the difference operator at the level of the pixel data between the time points *t* and t+τ. This operation produces a novel image with the existing movement during this moment, which is considered a temporal sequence. Mathematically, temporal features are formulated in Equation (2):

(2) $$g_{d,t}(x,y)=[g_{t+\tau }(x,y)-[g_{t}(x,y)]$$

In this study, the time interval between two selected frames is τ=1. Furthermore, we performed a second step of extraction of spatio-temporal features from the dataset, and this was realized using the deep neural network EfficinetNet-B0. This model is applied to both spatial and temporal images, functioning as an extraction mechanism with the new data sequences. EfficientNet [41] is a highly effective compound scaling method that uniformly scales all dimensions of depth/width/resolution using a simple yet highly effective compound coefficient. All EfficientNet models from B0 to B7 are scaled from our baseline EfficientNet-B0 using a different compound coefficient that is trained on more than one million images from the ImageNet database. In the end, we concatenated the numerical outputs, and then we obtained a feature vector ready to be refined and regularized to predict the test samples.

### 3.2 Transfer learning model and fine-tuning

Transfer learning helps transfer knowledge from a trained machine learning model to a different but related problem. In other words, it transfers the weights that a network has learned at the first task to the new one. The combination with neural networks has become quite advantageous in saving training time, reducing computation costs, getting usually the better performance of neural networks, and not needing a lot of data. In the present work, we had the benefit of this already-acquired knowledge from the EfficientNet-B0 model that was trained on the ImageNet dataset, which is large enough to create a generalized model (1.2 million images). Thus, precise adjustments made to parameters could also enhance accuracy, as mentioned in Table 1.

**Table 1:** Model tuning parameters.

Parameter	Selected value
Video length	10 seconds
Input size	224 × 224 × 3
Color channel	3 (RGB)
Epochs	100
Batch size	64
Dropout	0.2 - 0.4
Weights	ImageNet
Bias initializer	Zeros
Kernel initializer	he_uniform
Optimizer	Adam
Learning rate	0.001
Loss function	Categorical cross_entropy
Activation function	Relu - Softmax

**Figure 2:**
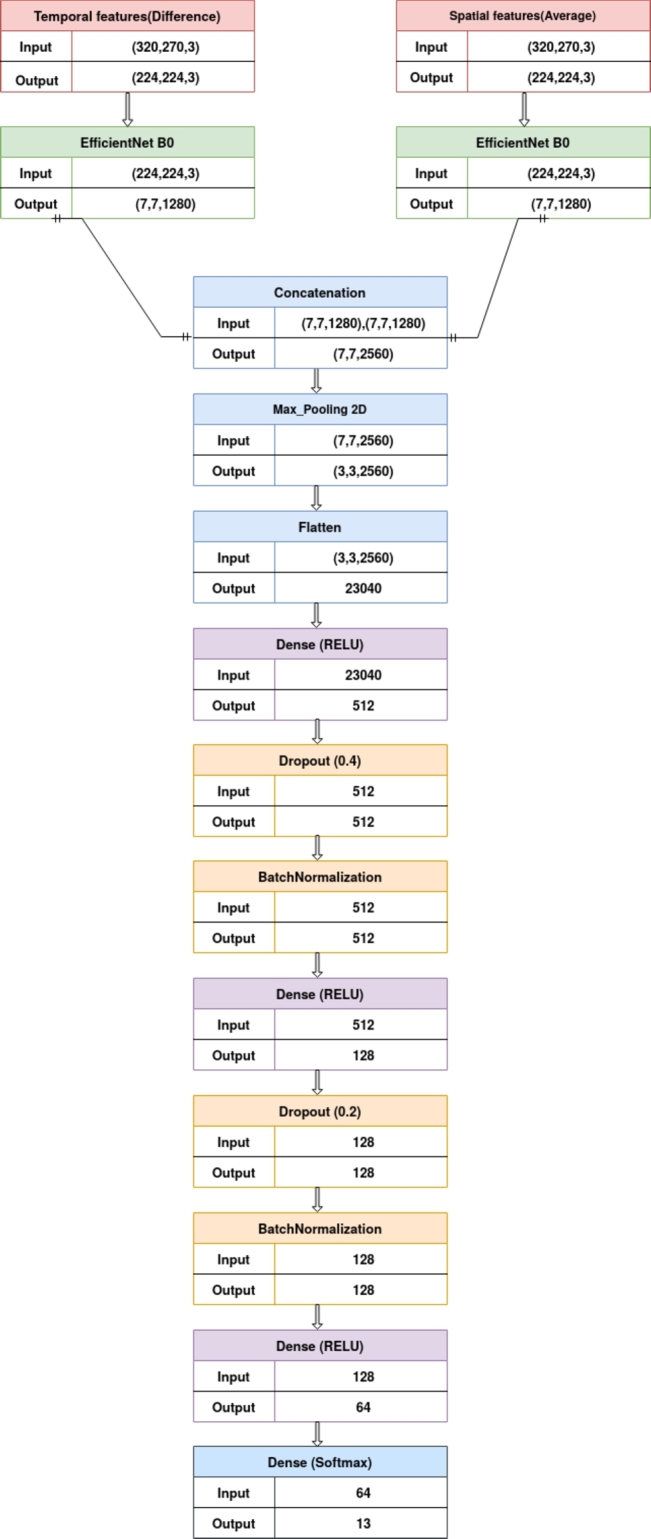
The visualization of the proposed SPEFF model on the Yuppen dataset.

During the implementation of our model, we noticed overfitting even though various preventative measures were taken. Therefore, an ample amount of adjustments and regularization in different forms is needed [121]. Consequently, we performed a fine-tuning process for the pre-trained model. There are different ways to fine-tune a model by considering two major factors: data size and data similarity. These include fine-tuning all or some parameters of the last few layers of a pre-trained model, like training some layers while freezing others, or using a pre-trained model as a feature extraction mechanism. In our particular case, during the training stage, we kept the pre-trained model EfficientNet-B0 without any change, but we modified the output dense layers by adding new ones, and we integrated more max-pooling, dense, batch-normalization, and dropout layers (i.e., the regularization phase of numerical data) at the end of our proposed architecture. Hence, the EfficientNet-B0 is used as an extraction mechanism where the output layer is removed (the one that gives the probabilities) and the entire network is considered a fixed feature extractor for a dataset. Thus, we removed the dense output layer for 1,000 classes and replaced it with max-pooling 2D, followed by a dense output layer (512, RELU), dropout (dropout probability of 40%), a batch-normalization layer, a dense layer (128, RELU), dropout (dropout probability of 20%), batch-normalization layer, a dense layer (64, RELU), and the dense output layer for thirteen classes for the Yupenn dataset. The added layers are illustrated in the architecture presented in Fig. 2. The complementary temporal information is then combined with spatial features to finally determine the output class label of dynamic textures. The learning rate is fixed at 0.001; this is one of the key hyperparameters that scales the magnitude of our weight updates to minimize the network's loss function. The Adam optimizer is used as a gradient descent algorithm in our proposed STEFF model. Table 1 shows the model tuning parameters. The following section discusses the model performance of the STEFF method.

## 4 Training and performance

Performance evaluation can help to define areas of the predictive model that need improvement and determine whether the program is achieving its goals or objectives. However, there are no standardized methods for evaluating the effectiveness of any deep convolutional neural network model. Table 1 explains the model tuning parameters and the following subsections describe the computing hardware, the used framework, and the evaluation metrics.

### 4.1 Model training and hardware implementation

The model was constructed and trained on the training dataset with Python using an HP Z620 workstation with an NVIDIA GeForce GTX 1050 Ti GPU card with 48 gigabytes (GB) of memory (RAM), an Intel(R) Xeon(R) Central Processing Unit (CPU) E5-2670, and a processor base frequency of 2.60 GHz. Moreover, the Python programming language and Keras framework are used for the code implementation of the dynamic texture classification model.

### 4.2 Model tuning parameters

The overfitting problem was the major challenge faced in this implementation on the large architecture, and thus various model tuning parameters have to be carefully chosen using insights into the execution time and complexity of the proposed model that should be optimally efficient. This configuration includes dropout layers, batch normalization layers, and several dense layers.

• Number of epochs (100): a hyperparameter that defines the number of times that the full training dataset will be processed by the learning algorithm.
• Batch size (64): the number of processed images that will be propagated through the network.
• Learning rate (0.001): the step size while searching for the minimum value of the loss function at each iteration.
• Optimizer (Adam): is a stochastic gradient descent algorithm that minimizes the loss function for training DL models.
• Activation Function (ReLU): A linear unit is a mathematical function applied to a signal at the output of an artificial neuron that is computationally efficient.
• Bias initializer (Zeros): which is a parameter that determines how the biases are first set before training the model.
• Kernel initializer (he_uniform): is a function of statistical distribution for initializing random weights of layers.
• Loss function (Categorical cross-entropy): evaluation method that determines how well your algorithm models your dataset.
• The pooling layer (Max Pooling2D): is used to decrease the size of sampling feature maps (input images) to reduce the number of computations in the network. The training will therefore be faster.
• Dropout: is a technique to reduce overfitting when training the model.

Table 1 represents all details of our model tuning parameters used in our experiments.

### 4.3 Evaluation metrics

This section describes the evaluation metrics used to evaluate model performance during training and testing. Classification metrics include accuracy, precision, recall, the F1 score, the AUC-ROC value, and the confusion matrix. The formulas that define how these metrics were calculated are in the equations below (Eq. (3), Eq. (4), and Eq. (5)).

(3) $$Precision=\frac{TP}{TP+FP}$$

(4) $$Recall=\frac{TP}{TP+FN}\;.$$

(5) $$F1score=2\times \frac{Precision\times Recall}{Precision+Recall}\;.$$

Where TP, TN, FP, and FN represent true positives, true negatives, false positives, and false negatives, respectively. AUC-ROC is the area under the receiver operating characteristics curve that provides a probability, meaning the score value is between 0 and 1. It is one of the most important evaluation metrics for identifying any classification model's performance. We applied the one-versus-all technique to obtain a weighted AUC-ROC score for our model. Also in the same context, the confusion matrix achieves a similar goal by defining and visualizing the performance of a classification algorithm. On the other hand, there are two other performance measurement factors: the accuracy score using 5-fold Cross-Validation and the timing of implementation for each image classification. Increasing accuracy rates and decreasing execution times are the main objectives that any deep learning model tries to achieve.

## 5 Experiments and evaluations

In this section, we evaluate the proposed approach using the existing outdoor video datasets. We use three types of datasets related to dynamic texture and dynamic scene, namely, DynTex++, Yupenn, and Yupenn++, which are described in the following subsection. We performed both quantitative and qualitative experiments.

### 5.1 Used datasets

A detailed description of datasets frequently used for dynamic texture classification is given in Table (5). In this study, our research primarily centers on the domain of outdoor scene recognition, which poses unique challenges in dynamic texture classification. To align with the scope of our research and the specific challenges posed by outdoor scenes, we deliberately focused our evaluation on a subset of categories within the datasets used.

For the DynTex++ dataset, we conducted evaluations on 14 selected categories out of the total 36 categories available. Similarly, in the case of the Yuppenn dataset, our evaluations concentrated on 13 chosen categories out of 14. For the Yuppenn++ dataset, we assessed our approach using 18 specific categories out of 20 available (refer to Table 2, Table 3 and Table 4). The pink cells to indicate outdoor categories, which form the primary focus of our study, encompassing the outdoor scenes found within the Yupenn dataset. In contrast, the green cells denote the remaining categories within the dataset, which are predominantly composed of artificial motion scenes. This color differentiation serves to underscore the specific emphasis of our research on outdoor and natural scenarios. This category selection was made purposefully to ensure that our research addresses the nuances and complexities of outdoor scene dynamic texture classification.

**Table 2:**
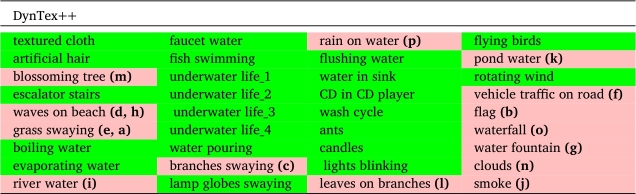
Scene categories in the DynTex++ dataset.

**Table 3:**
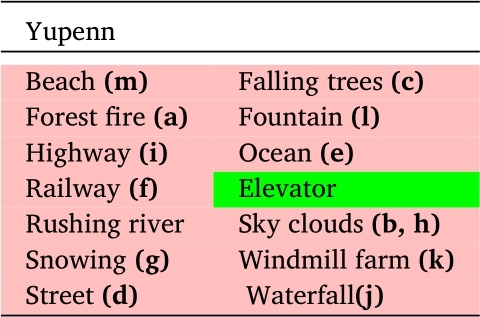
Scene categories in the Yupenn dataset.

**Table 4:**
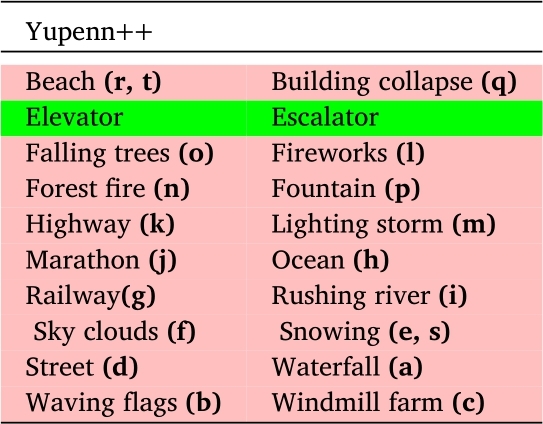
Scene categories in the Yupenn++ dataset.

**Table 5:** Summary of texture natural datasets used in our experiments.

Dataset	Texture classes	Videos in total	Samples in total	Sample size (Pixels)
Dyntex++	14	168	33,600	352×288
Yupenn	13	390	56,152	320×270
Yupenn++	18	540	76,475	320×270

#### 5.1.1 DynTex++ dataset

**Figure 3:**
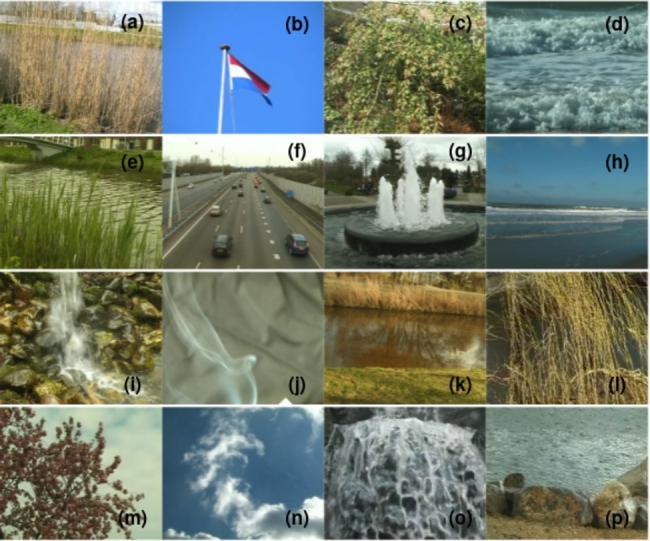
Sample frames from different classes of the DynTex++ dataset. Each sub-figure represents a specific class from (a) to (p).

The sequences in the DynTex dataset are reorganized to provide DynTex++ [107], a more robust benchmark for DT recognition. As a result, the 345 raw videos of DynTex are divided into sub-sequences with a defined size of 50×50×50, so that they only contain the primary dynamic texture and exclude any backgrounds or additional dynamic structures. The 3,600 sequences that were revealed by the filtered clipped DTs were then grouped into 36 categories with 100 DTs for each, and we used only fourteen classes that contain natural outdoor scenes, then transformed them into 33,600 frames in total, with a dimension of 352×288. Fig. 3 represents the selected samples from the DynTex++ dataset, Each sub-figure corresponds a distinct class labeled from **(a)** to **(p)**, and detailed descriptions are available in Table 2.

#### 5.1.2 Yupenn dataset

**Figure 4:**
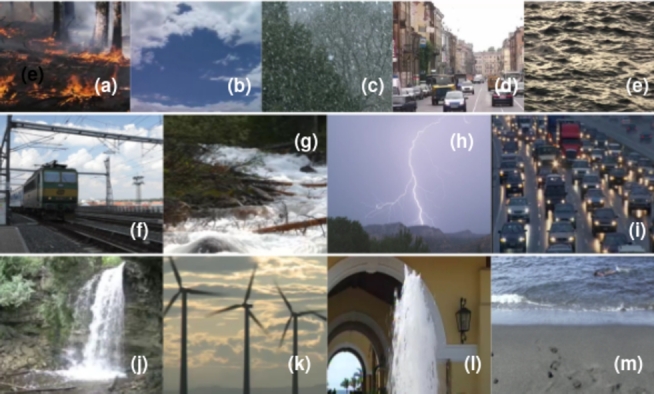
Sample frames from different classes of the Yupenn dataset. Each sub-figure represents a specific class from (a) to (m).

The Yupenn stabilized dynamic dataset [110] is used to evaluate the classification performance of the various orientation-related representations and to emphasize scene-specific temporal information. It is composed of 14 natural scene categories containing 30 color videos with an average of 145 frames per scene. We used 13 natural outdoor classes in our work, which are moved into 56,152 frames with a dimension of 320×270. Fig. 4 depicts the selected samples from the Yupenn dataset, Each sub-figure corresponds a distinct class labeled from **(a)** to **(m)**, and detailed descriptions are available in Table 3.

#### 5.1.3 Yupenn++ dataset

**Figure 5:**
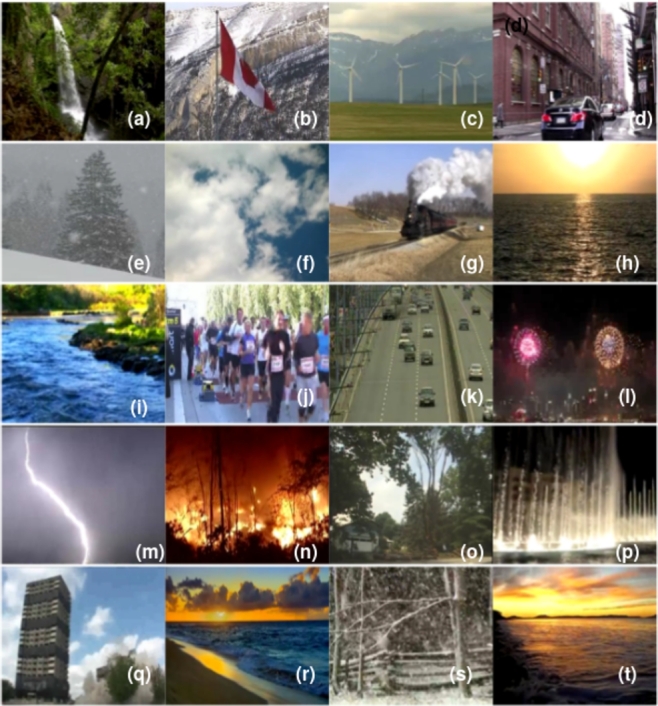
Sample frames from different classes of the Yupenn++ dataset. Each sub-figure represents a specific class from (a) to (t).

Six more classes, for a total of 20, have been added to the original Yupenn dataset. The following categories are finally included in the dataset: beach, city street, elevator, forest fire, fountain, highway, lightning storm, ocean, railway, rushing river, clouds, snowing, waterfall, wind-mill farm, building collapse, escalator, falling trees, fireworks, marathon, and waving flags. The last six listed classes are in addition to those available in the earlier Yupenn. This innovative dataset is known as YUpenn++ [122] since it has a larger number of classes and moving camera videos. There are 60 color videos in the dataset for each scene class, with no two samples for a given class taken from the same physical scene. We focused on 18 outdoor classes that contain 76,475 frames with a dimension of 320×270. In Fig. 5, selected samples from the Yupenn++ dataset are showcased. Each sub-figure corresponds to a distinct class labeled from (a) to (t), with detailed descriptions in Table 4.

### 5.2 Experimental results and discussion

The main objective of the STEFF model is to classify dynamic textures. For this purpose, we performed a dual combination of temporal and spatial features, that were extracted passing through two phases. The first occurs when the difference and average operations have been calculated between the pixels of successive frames, generating, in fact, a new modified image with motion and appearance information separately. At this level, the next phase materializes in the field of neural networks, where new features were again extracted from these sequences with the pre-trained model EfficientNet-B0 using the Python programming language. Then we began concatenating features, fine-tuning, and regularizing our model. The detailed architecture is presented in Fig. 2.

#### 5.2.1 Overall performance of the proposed approach

To evaluate this methodology, we thought that it would be helpful to compare the result with other models, saving the same architecture but using different pre-trained models, namely ResNet50, VGG16, and DenseNet121. We created 16 combinations with the four pre-trained models presented as the “A_B” approach, where “A” refers to the model that will be applied to temporal features generated from phase 1, and “B” is the model that will be applied to spatial features extracted from phase 1. We note that the Eff_Eff approach is referred to as STEFF which is our proposed method, the spatio-temporal EfficientNet. Table 6 exhibits the accuracy rate, loss score, and inference time for one image, as well as the precision, recall, F1 score, and AUC-ROC results of the combined methods “A_B” assessed on the Yupenn dataset. The related train and test accuracies for each iteration were noted. To assess the performance and generalization ability of our model, we used 5-fold cross-validation. We calculated the mean value over the five iterations to efficiently describe accuracies as presented in Table 6. By using this method on all datasets, we aim to present a consolidated measure of the model's performance that accounts for how the model performs across the different folds.

**Table 6:** The performance comparison between our proposed spatio-temporal and transfer learning models.

Spatio_ Temporal Models	Execution Time for one image (Second)	Testing Accuracy % Epochs = 100	Testing Loss Epochs = 100	Precision	Recall	F1 score	AUC ROC
Eff_Temporal	0.68	92.77	0.39	__	__	__	__
Eff_Spatial	0.66	95.58	0.20	__	__	__	__

**Eff_Eff(STEFF)**	**0.70**	**97.34**	**0.10**	**98.00**	**97.00**	**97.00**	**1**
Eff_Vgg	3.41	97.01	0.14	97.00	97.00	97.00	1
Eff_Res	2.34	81.62	1.90	83.00	82.00	81.00	0.97
Eff_Den	2.21	89.46	0.96	90.00	90.00	89.00	0.99

Den_Eff	2.28	82.17	1.80	83.00	82.00	82.00	0.96
Den_Vgg	4.56	77.92	1.48	78.00	78.00	78.00	0.98
Den_Res	3.34	66.52	5.06	67.00	67.00	66.00	0.87
Den_Den	1.76	85.90	1.89	86.00	87.00	86.00	0.94

Res_Eff	2.27	86.61	1.45	88.00	87.00	87.00	0.98
Res_Vgg	4.55	83.76	1.67	85.00	84.00	83.00	0.96
Res_Res	1.76	87.89	1.75	89.00	89.00	88.00	0.96
Res_Den	3.20	66.19	4.74	68.00	67.00	66.00	0.89

Vgg_Eff	3.46	95.73	0.17	96.00	96.00	96.00	1
Vgg_Vgg	3.10	96.58	0.13	97.00	97.00	97.00	1
Vgg_Res	4.58	85.88	1.41	87.00	86.00	86.00	0.98
Vgg_Den	4.57	75.93	1.68	77.00	76.00	76.00	0.97

a **Eff_Temporal**: EfficientNet on temporal features. b **Eff_Spatial**: EfficientNet on spatial features. c **STEFF**: Spatio-temporal efficientNet. d **Eff**: EfficientNet_B0. e **Den**: DenseNet_121. f **Res**: ResNet50. g **Vgg**: VGG16.

**Figure 6:**
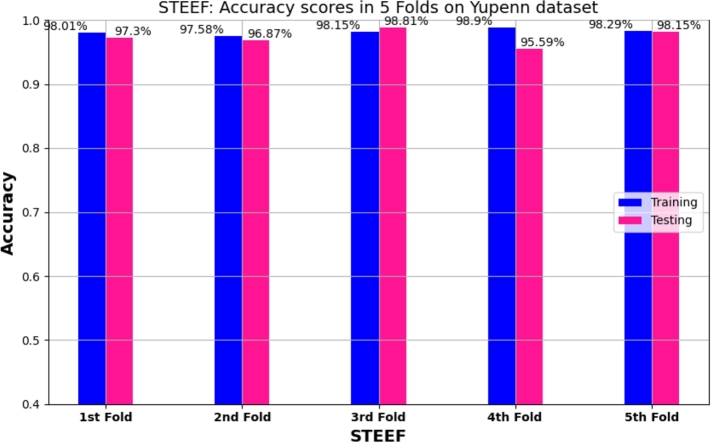
5 Folds cross-validation results: training and testing accuracy on Yupenn dataset for STEFF approach.

We presented in Fig. 6 a bar chart showcasing the accuracy results for different folds obtained from our proposed STEFF model. The chart illustrates the training and testing accuracies for each fold, with the accuracy values displayed on top of the bars.

**Figure 7:**
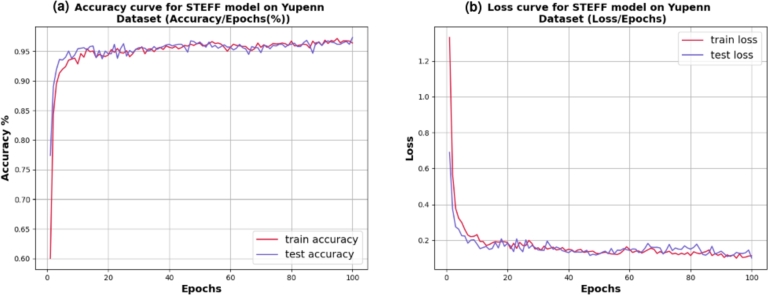
Performance evaluation of the STEFF model on the Yuppen dataset. (a) Accuracy plot. (b) Loss plot for both training and testing phases.

**Figure 8:**
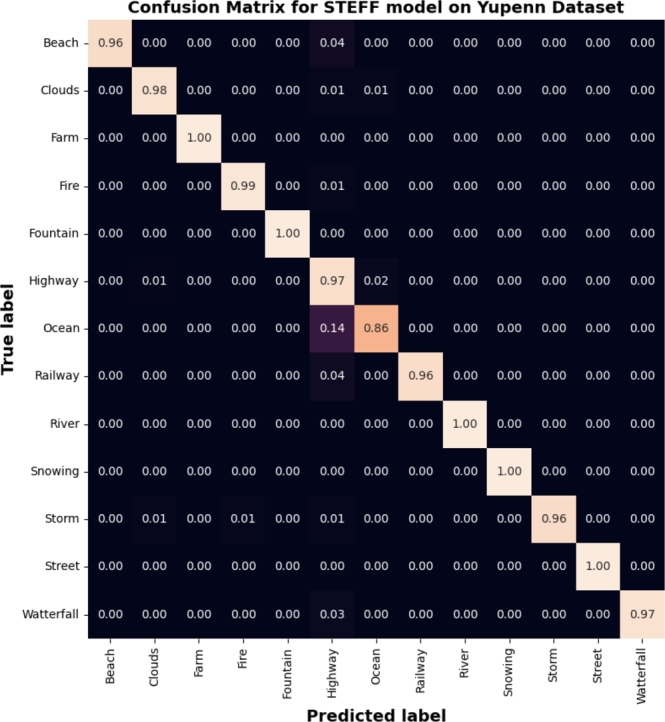
Confusion matrix resulted from the STEFF model on the Yuppen dataset.

Various ensemble classifiers were run as shown in Table 6. The accuracy rates of ensemble classifier models ranged between 66.19% and 97.34%. The combined model Eff_Eff known as STEFF obtained the highest average accuracy (i.e., 97.34%) and the lowest loss score (0.10), which corresponds to the infernal time for one image of 0.75 seconds when comparing with the 16 combined CNN models for the number of epochs of 100. Fig. 7 represents the accuracy **(a)** and loss **(b)** between the training and testing phases, obtained from our proposed STEFF model. Initially, the mean accuracy starts at a training rate of 60%, then it constantly increases up to 97% accuracy. It also shows validation accuracy. Initially, the mean testing accuracy starts at 77.50%, which has a variable increment in accuracy. It constantly increases by approximately 97.34% in accuracy. The loss starts training value of 1.3 and a testing value of 0.7, and then both curves converge and tend toward 0.10. According to the simulation results, the proposed framework achieves precision, recall, and an F1 score of 98, 97, and 97 percent, respectively, as well as an AUC-ROC score of 1. In addition, Fig. 8 illustrates the confusion matrix of the STEFF model based on the testing data.

Furthermore, when we extracted temporal features with EfficinetNet-B0 and spatial features with the pre-trained model VGG16, we noticed an approximate accuracy of 97.01% and a loss of 0.14, but computationally more complex and expensive in terms of timing, which estimated about 3.41 seconds for recognizing one image. Based on the results shown in Table 6, the composition of VGG16 and EfficientNet-B0 provides satisfying and better outcomes, and even if we inverse their spatial and temporal aspects, Eff_Vgg to Vgg_Eff, we, therefore, save approximately the same comportment. Moreover, in this research, we combined DenseNet121, ReseNet50, EfficientNet-B0, and VGG16 in an arrangement of two pre-trained models “A_B”. The worst results were the Res_Den and Den_Res with an accuracy rate of 66.19% and 66.52%, respectively. In addition, we ran two other experiments in which we applied the EfficientNet-B0 to the same Yupenn dataset but only to the temporal frames generated in the first phase of the spatial sequences in the second experiment. In this order, we got an accuracy of 92.77%, and 95.58%, respectively.

**Figure 9:**
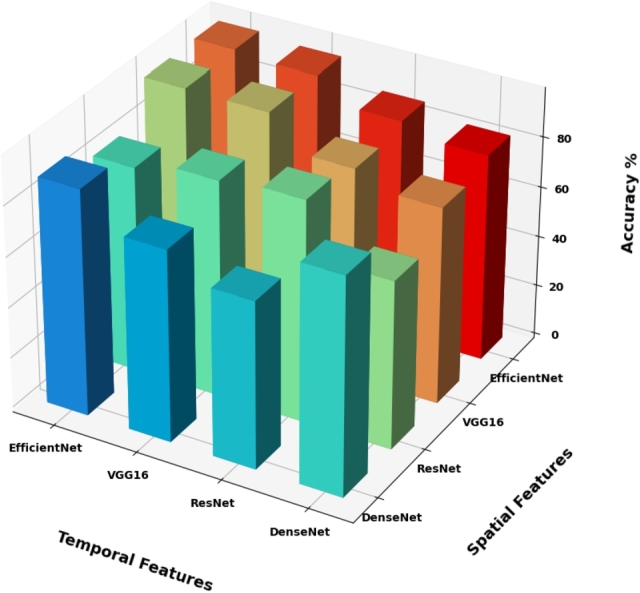
The performance comparison of proposed spatio-temporal models on the Yuppen dataset.

The analysis results confirm that the proposed model performs better than other models in terms of accuracy, loss, inference time, precision, recall, F1 score, and AUC-ROC, as presented in detail in Table 6 and visualized in a 3D diagram in Fig. 9.

To validate the effectiveness of our proposed approach on outdoor scene videos, we evaluated the model on the DynTex++ and Yupenn++ datasets using 5-fold cross-validation. Table 7 summarizes the performance of STEFF in terms of testing accuracy, testing loss, and timing for pretending the class of a single frame.

**Table 7:** The performance of our proposed STEFF model on different datasets.

Spatio_ Temporal Model	Execution Time for one image (Seconds)	Testing Accuracy (%) Epochs = 100	Testing Loss Epochs = 100	Dataset
STEFF	0.67	99.34	0.01	DynTex++
STEFF	0.70	97.34	0.10	Yupenn
STEFF	0.69	98.90	0.04	Yupenn++

With the same experience steps and parameters, we found the following results:

• On the DynTex++ dataset with 14 classes, the STEFF achieves an accuracy of 99.34% with a loss of 0.01, classifying one single image in 0.67 seconds.
• On the Yupenn dataset with 13 classes, the STEFF achieves an accuracy of 97.34% with a loss of 0.10, classifying one single image in 0.70 seconds.
• On the Yupenn++ dataset with 18 classes, the STEFF achieves an accuracy of 98.90% with a loss of 0.04, classifying one image in 0.69 seconds.

**Figure 10:**
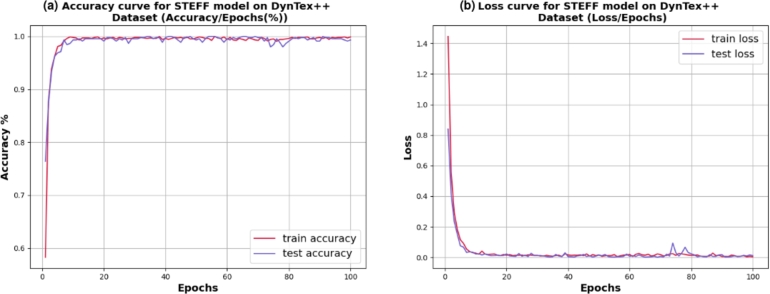
Performance evaluation of the STEFF model on the DynTex++ dataset. (a) Accuracy plot. (b) Loss plot for both training and testing phases.

**Figure 11:**
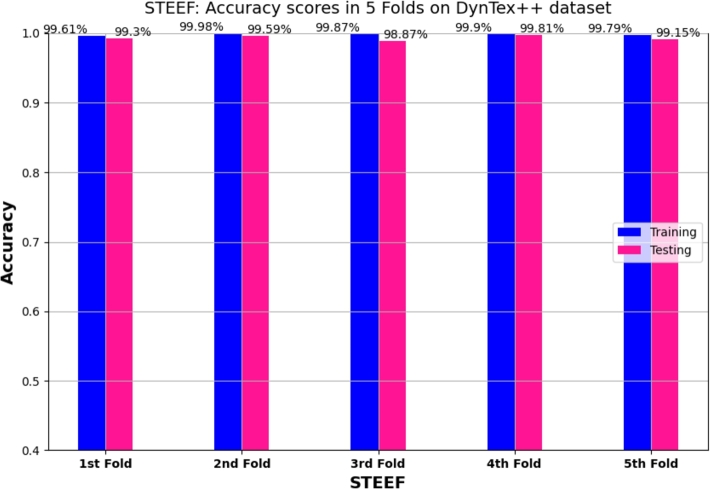
Cross-validation results: training and validation accuracy on DynTex++ dataset for 5 folds of STEFF approach.

**Figure 12:**
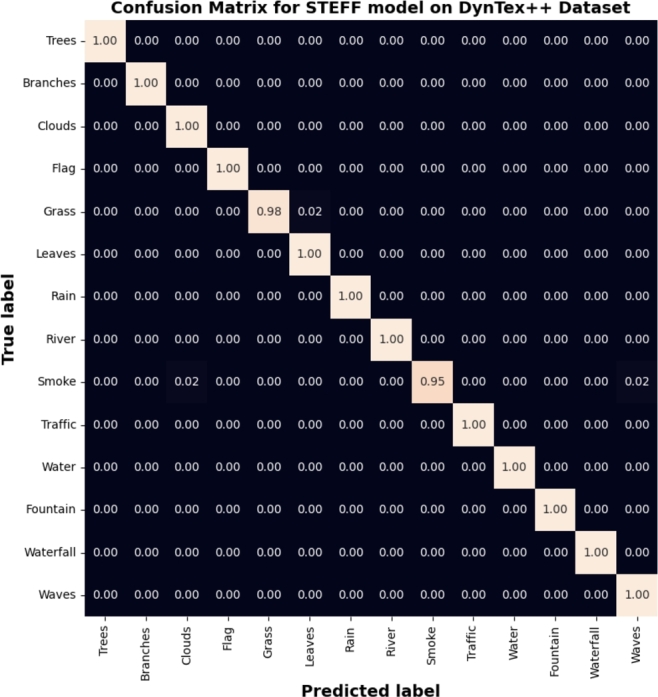
Confusion matrix resulted from the STEFF model on DynTex++ dataset.

Fig. 10 represents the line graph of outdoor model accuracy **(a)** and loss **(b)** of the STEFF approach for training and testing on the DynTex++ dataset. This process continues for up to 100 epochs. Initially, it starts training accuracy at 68% and testing accuracy at 76%. Then the two curves constantly increase, reaching 99.34%. Thus, the loss curve starts with training data approximately at 1.3 and testing samples at 0.85, then tends to 0.01. Additionally, the 5-fold cross-validation result of the training and testing accuracy on DynTex++ is illustrated in Fig. 11, while Fig. 12 presents the confusion matrix of the testing DynTex++ dataset.

**Figure 13:**
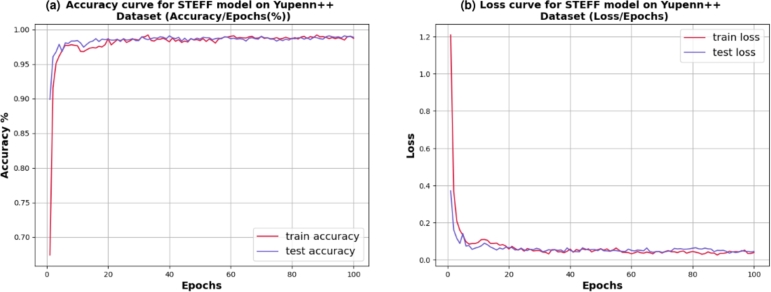
Performance evaluation of the STEFF model on the Yuppen++ dataset. (a) Accuracy plot. (b) Loss plot for both training and testing phases.

**Figure 14:**
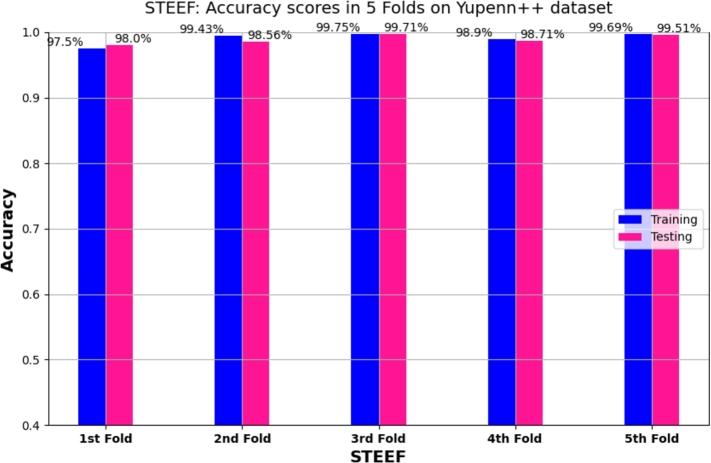
Cross-validation results: training and validation accuracy on Yupenn++ dataset for 5 folds of STEFF approach.

**Figure 15:**
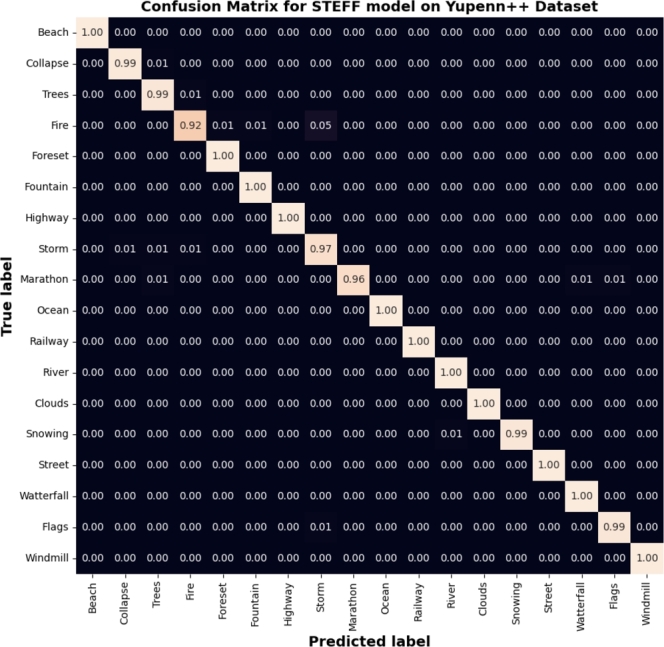
Confusion matrix resulted from the STEFF model on the Yuppen++ dataset.

Also, Fig. 13 illustrates the accuracy **(a)** and loss **(b)** plot for training and testing on the Yupenn++ dataset. This process continues for up to 100 epochs. It begins with a training accuracy of 67% and a testing accuracy of 90%, then gradually increases to 98.90%. Thus, the loss curve starts with training data approximately at 1.2 and testing samples at 0.36, then tends to 0.04. In addition, the 5-fold cross-validation result of the training and testing accuracy on Yupenn++ is illustrated in Fig. 14, Fig. 15 shows the confusion matrix of the testing Yupenn++ dataset.

Furthermore, to show the prediction results of the STEEF approach among the different classes, the classification confusion matrix is presented in Figure 8, Figure 12, Figure 15, where it should be noted that the STEFF approach achieves a good performance in all the scene categories of the three datasets except ocean, where 14% of frames are misclassified as a highway in Yupenn, also smoke class where 5% of sequences are misclassified as waves in Dyntex++ and finally, fire class where 8% of frames are misclassified as forest, fountain, and storm in Yupenn++. However, the proposed method obtains a superior performance on the other classes. Thus, from the experiments with the three datasets, it can be seen that the proposed STEFF method can also obtain a satisfactory network architecture that is suitable for outdoor scene recognition.

#### 5.2.2 Comparison with state-of-the-art

This section provides the comparison between the proposed approach and the state-of-the-art approaches that have previously shown great performance in dynamic scene recognition, which are demonstrated in Table 8, Table 9, Table 10.

**Table 8:** Comparison between proposed method and state-of-the-art approaches on DynTex++ dataset.

N°	Encoding methods	Accuracy rate (%)	N°	Encoding methods	Accuracy rate (%)
1	*DL* − *PEGASOS*^⁎^[107]	63.70${}^{⁎S}$	27	DDLBP with MJMI [102]	95.80
2	DFS [88]	91.70	28	HILOP [48]	96.21
3	*V* − *DFS*[88]	68.22	29	CVLBC [86]	91.31^*N*^
4	*S* − *DFS*[88]	88.71	30	*PCA* − *cLBP*[73]	91.90^*N*^
5	*FD* − *MAP*[96]	95.69	31	*PCA* − *cLBP*[73]	92.40^*S*^
6	3*D* − *OTF*[90]	89.17^*S*^	32	VLBP [20]	94.98^*N*^
7	WMFS [89]	88.80^*S*^	33	HLBP [98]	96.28^*N*^
8	STLS [91]	94.50	34	*MPCAF* − *TOP*[86]	96.52^*N*^
9	FDT [96]	95.31	35	*CLSP* − *TOP*[101]	95.50^*N*^
10	DNGP [70]	93.80	36	*ASF* − *TOP*[72]	95.40^*N*^
11	KGDL [93]	92.80^*S*^	37	*LBP* − *TOP*[20]	94.05^*N*^
12	OTDL [94]	94.70^*S*^	38	*LPQ* − *TOP*[79]	95.00^*N*^
13	EKDL [95]	93.40^*S*^	39	*MBSIF* − *TOP*[87]	97.12^*N*^
14	DDTP [97]	95.09	40	$HoGF_{\left\{\sigma =1\};\{2nd,3rd\right\}}^{2D}$[106]	97.19
15	NLSSA [92]	92.40^*S*^	41	$HoGF_{\left\{\sigma =1\};\{3rd,4th\right\}}^{3D}$[106]	97.63
16	KSSA [92]	92.20	42	$DoDGF_{\left(0.7,1);\{1st\right\}}^{2D}$[47]	96.40
17	DKSSA [92]	91.10	43	$DoDGF_{\left(0.7,1);\{1st,2nd\right\}}^{2D}$[47]	97.14
18	*MMDP*_*D*_*M*/*C*_[51]	95.86	44	$DoDGF_{\left(0.7,1);\{1st\right\}}^{3D}$[47]	97.15
19	*MEMDP*_*D*_*M*/*C*_[51]	96.03	45	$DoDGF_{\left(0.7,1);\{1st,2nd\right\}}^{3D}$[47]	97.52
20	MEWLSP [105]	98.48^*N*^	46	*SOE* − *NET*[111]	94.40^*S*^
21	WLBPC [100]	95.01^*N*^	47	*DT* − *CNN* − *AlexNet*[109]	98.18^⁎^
22	FoSIG [99]	95.99	48	*DT* − *CNN* − *GoogleNet*[109]	98.58^⁎^
23	*V* − *BIG*[104]	96.65	49	*B*3*DF* − *S*[68]	94.80^*N*^
24	RUBIG [65]	97.08	50	*B*3*DF* − *SM*[68]	95.90^*N*^
25	High level feature [108]	69.00^*S*^	51	*B*3*DF* − *SMC*[68]	95.58^*N*^
26	Chaotic vector [85]	69.00	52	**Our STEFF(All-categories)**	**98.53**^⁎^

**Note**: “-” means “not available”. The superscript “S” stands for SVM, and “N” stands for NN. Superscript “*” indicates results using deep learning algorithms.

**Table 9:** Comparison between proposed method and state-of-the-art approaches on Yupenn dataset.

N°	Encoding methods	Accuracy rate (%)	N°	Encoding methods	Accuracy rate (%)
1	GIST [123]	56.00^*N*^	26	*Hybrid* − *CNN*[117]	98.10
2	HOF [124]+ GIST [123]	68.33^*S*^	27	*LTP* − *TOP*[59]	86.40
3	Chaos [54]+ GIST [123]	22.86^*S*^	28	VLBP [20]	81.70
4	*SOE*^*S*^[110]	80.71^*S*^	29	2*DHF* − *LBP* − *TOP*[58]	91.70
5	*SOE*^*N*^[110]	74.00^*N*^	30	*HOG* − *TOP*[64]	86.80
6	SFA [74]	85.48^*S*^	31	*Single* − *Frame* − *CNN*[119]	87.00^⁎^
7	CSO [80]	85.95^*S*^	32	3D pyraNet-F [120]	93.60^⁎^
8	BoSE [75]	96.19^*S*^	33	LSTF [67]	98.00
9	C3D [112]	87.70^*S*^	34	*st* − *TCoF*[114]	99.05^*S*^
10	DPCF [76]	99.00	35	*st* − *TCoF*[114]	98.81^*N*^
11	SA-CNN [125]	98.33^⁎^	36	*SK* − *means*[82]	95.20^*S*^
12	BoST [77]	85.47^*S*^	37	ResNet [126]	91.90^⁎^
13	LBP-TOP [20]^*S*^	85.29^*S*^	38	VGG	97.40${}^{S⁎}$
14	LBP-TOP [20]^*N*^	75.95^*N*^	39	CSR [127]	94.00
15	*V* − *SAP*^*S*^[103]	94.05^*S*^	40	Holistic approach [83]	97.00
16	*V* − *SAP*^*N*^[103]	87.86^*N*^	41	Region based approach [83]	90.40
17	SAE^*S*^[82]	96.00^*S*^	42	*DLTP* − *TOP*^*S*^[83]	89.20
18	SAE^*N*^[82]	80.70^*N*^	43	*DLTP* − *TOP*^*CNN*^[83]	97.00^⁎^
19	Imagenet [115]	96.70	44	*DLTP* − *TOP*^*RF*^[83]	83.60
20	DDM [71]	97.52	45	$CSAP-TOP_{a}^{S}$[103]	90.00^*S*^
21	DDM+SCSP[71]	**99.18**	46	$CSAP-TOP_{a}^{N}$[103]	81.67^*N*^
22	Bi-CNN [116]	99.00^⁎^	47	$CSAP-TOP_{b}^{S}$[103]	94.76^*S*^
23	SIFT+5DMFV [56]	85.61	48	$CSAP-TOP_{b}^{N}$[103]	86.67^*N*^
24	D3 [118]	99.05	49	E-SVM [46]	96.43
25	*D*3_*d*_[118]	98.33	50	**Our STEFF(All-categories)**	$98.42^{⁎}$

**Note**: “-” means “not available”. The superscript “S” stands for SVM, and “N” stands for NN. Superscript “*” indicates results using deep learning algorithms.

**Table 10:** Comparison between proposed method and state-of-the-art approaches on Yupenn++ dataset.

N°	Encoding methods	Accuracy rate (%)
1	SFA [74]	56.90
2	BoSE [75]	77.00
3	IDT [128]	85.60
4	C3D [112]	84.00
7	ResNet-50	85.90*
8	T-ResNet	89.00*
9	Attention matrix [113]	92.00
10	Single attention [113]	88.00
11	ATP-Net [113]	92.30*
12	**Our STEFF(All-categories)**	98.56*

**Note**: The Superscript “*“indicates results using deep learning algorithms.

Hereinafter, we detail evaluations of STEFF's performances for all three datasets [110], [107], [122].

On the DynTex++ dataset, our method achieved an impressive recognition rate of 98.53%, outperforming many state-of-the-art methods presented in the table. Notably, this rate positions it among the top-performing methods listed in Table 8 across multiple categories, including optical flow-based, model-based, geometry-based, filter-based, local feature-based, and learning-based approaches.

Among the deep learning methods, MEWLSP [105] stands out with an accuracy of 98.48% using the NN classifier. Additionally, DT-CNN-AlexNet [109] and DT-CNN-GoogleNet [109] achieved accuracies of 98.18% and 98.58%, respectively. It's worth noting that MEWLSP employed a high-dimensional DynTex description, while DT-CNN-AlexNet and DT-CNN-GoogleNet utilized complex deep learning algorithms, resulting in longer programming times.

In Table 9, the highest performance achieved on Yupenn is 99.18%, as obtained by the Deep Discriminative Model (DDM) when combined with Sparse Cubic Symmetrical Pattern (SCSP), resulting in the DDM+SCSP method [71]. Notably, this performance surpasses that of our STEFF model by approximately 0.76%. Specifically, DPCF, D3, Bi-CNN, st-TCoF when coupled with both KNN and linear SVM classifiers, achieved improvements of approximately 0.58%, 0.63%, 0.58%, 0.39%, and 0.63% compared to our approach due to their discriminative power. These differences are considered relatively minor, as our STEFF model still ranks among the top ten methods presented in the list. Additionally, our proposed solution demonstrated superior performance compared to existing dynamic texture descriptors, such as SA-CNN, $D3_{d}$, LSTF, Hybrid-CNN, and DDM, with improvements of approximately 0.09%, 0.09%, 0.42%, 0.32%, and 0.9%, respectively. This includes Model-based categories, such as GIST (approximately 41.42% lower), HOF+GIST (approximately 30.09% lower), Chaos+GIST (approximately 75.56% lower), where HOF and Chaos focus on temporal information in videos, and GIST describes appearance information. Furthermore, BoST achieved 85.47%, which is 12.95% lower, and SOE obtained only 74.00% with the NN classifier and 80.71% with the SVM classifier, representing approximately 24.42% and 17.71% lower performance than our method.

Moreover, our proposed framework exhibited significant improvements compared to various approaches in the Filter and Local feature-based group, encompassing LBP-TOP, 2DHF-LBP-TOP, VLBP, CSO, BoSE, HOG-TOP, SFA, SAE, V-SAP, CSAP-TOP, C3D, BoST, SIFT-5DMFV, LTP-TOP, 3D pyraNet-F, SK-means, CSR, DLTP, Learning-based approaches like ResNet, VGG and Single Frame-CNN, Holistic approach, and Region-based approach.

On the Yupenn++ dataset (see Table 10), STEFF achieves an accuracy of 98.56%, significantly outperforming the state-of-the-art methods. Specifically, the improvements over the attention matrix and ATP-Net are 92.00%, which is 6.56% lower than our approach, and 92.30%, approximately 6.26% lower than STEFF, respectively.

From Table 8, Table 9, Table 10, we can demonstrate that using deep learning algorithms performs better than the other classifiers due to the powerful convolutional neural network concept, while the SVM [129] classifier performed better than the K-nearest neighbors classifier. Likewise, the high performance on the three datasets demonstrates the great effectiveness of the proposed STEFF for dynamic scene classification.

## 6 Conclusion

In this paper, we introduce a combined spatio-temporal neural network framework, STEEF, designed to provide an evolutionary dynamic texture classification network for outdoor video datasets. This framework simultaneously extracts and integrates motion features with appearance features in two distinct phases. The first phase involves image pre-processing, including normalization and frame-by-frame video operations. In the second phase, deep learning techniques utilizing CNN models are employed for feature extraction. Our experimental results underscore the effectiveness of the STEEF algorithm, which achieved remarkable accuracy rates of 99.34%, 97.34%, and 98.90% on the outdoor scenes dataset, Dyntex++, Yupenn, and the Yupenn++ dataset, respectively. Notably, a comprehensive comparative analysis of the 16 implemented learning models unequivocally designates STEEF as the optimal choice for dynamic texture recognition. Our approach involves a relatively high computational cost during feature extraction, which is considered a limitation of this study. In our future research endeavors, we will concentrate on reducing the dimensionality of the extracted features to alleviate this computational burden while maintaining classification quality. Nevertheless, our efficient implementation consistently accelerates the feature extraction process, achieving a noteworthy speedup of 0.7 per image when compared to all models in the study. This consistent improvement in performance across diverse datasets underscores the effectiveness, robust identification capabilities, and high classification accuracy of our method in the context of dynamic texture classification.

## Funding

The authors did not receive support from any organization for the submitted work.

## CRediT authorship contribution statement

**Kaoutar Mouhcine:** Writing – review & editing, Writing – original draft, Formal analysis, Data curation, Conceptualization. **Nabila Zrira:** Writing – review & editing, Writing – original draft, Supervision, Formal analysis, Conceptualization. **Issam Elafi:** Resources, Project administration, Investigation. **Ibtissam Benmiloud:** Writing – review & editing, Writing – original draft, Software, Resources, Investigation. **Haris Ahmad Khan:** Writing – review & editing, Writing – original draft, Supervision, Methodology, Conceptualization.

## Declaration of Competing Interest

The authors declare that they have no known competing financial interests or personal relationships that could have appeared to influence the work reported in this paper.

## Data Availability

The datasets used in our study are publicly available data, and they can be accessed from the following source https://vision.eecs.yorku.ca/research/dynamic-scenes/ for Yupenn and Yupenn++ datasets, and http://dyntex.univ-lr.fr/ for DynTex++ dataset. We have complied with the terms and conditions of use specified by the data source, and all necessary citations and attributions have been included in our study.
